# Comment on “DKA-Induced Takotsubo Cardiomyopathy in Patient with Known HOCM”

**DOI:** 10.1155/2017/4786510

**Published:** 2017-11-22

**Authors:** John E. Madias

**Affiliations:** ^1^Icahn School of Medicine at Mount Sinai, New York, NY, USA; ^2^Division of Cardiology, Elmhurst Hospital Center, Elmhurst, NY, USA

I very much enjoyed reading the contribution by Gordon et al., published on April 3, 2017 [1], about a 66-year-old man with known history of hypertrophic cardiomyopathy (HOCM) who suffered diabetic ketoacidosis- (DKA-) triggered Takotsubo syndrome (TTS) in the setting of newly detected diabetes mellitus (DM). There have been 2 previously reported cases of TTS associated with DKA, mentioned by the authors as their references #4 and #6 and also summarized elsewhere [2], but that report is the 1st in the United States associating TTS, HOCM, and DKA, as they state [1]. The authors did a marvelous job in working up and managing the patient, considering the complex pathophysiology which could ensue in patients with TTS superimposed on HOCM with previously detected intraventricular gradient and systolic anterior motion of mitral valve, further compounded by the triggering pathology of new DM with complicated DKA. The authors discuss the importance of the DKA-induced increases in serum catecholamines [1, 3], although in patients with established DM a decrease in norepinephrine and epinephrine release by the autonomic sympathetic nervous system and the adrenals, respectively, is also reported [4]. Although it has been reported that patients with TTS have low prevalence of DM, in comparison with the general population [4], there is still a close association of TTS and DM; in a meta-analysis of all 32,809 patients collectively reported as case series in the international literature, 17% had DM, and in 1,083 patients individually reported, 10.2% had DM [4]. Probably the 10.2% reflects the actual TTS-DM association, since the 17% refers to large cohorts of patients, encompassing repeated reporting of expanding groups of patients with TTS from the same world research centers. The elevated HgbA1c (11.8%) suggests that DM was present for some time; however, the exact onset of DM cannot be ascertained in this patient. Perhaps the authors could supply some information, whether the patient continued to show evidence of DM after the recovery from TTS and whether he had evidence of peripheral neuropathy or other end-organ diabetic changes, which occasionally presage the emergence of chronic hyperglycemia of chemical DM. Indeed, a neurologic work-up in the reported patient with HOCD, DM, and TTS is indicated.

The authors state that “the resolution of TC with DKA treatment, seen as improvement of the ejection fraction and left ventricular motion improvement on echocardiogram, suggests a direct association between the two conditions” [1] and that “when DKA was treated and glucose levels were brought within normal range, repeat echo revealed an improved EF and normal ventricular motion and, therefore, overall resolution of TC” [1]. However, similar rapid recovery of the left ventricular function (LVF) (in a few days and indeed sometimes in a few hours) in patients presenting with TTS has been repeatedly reported in the literature, without being attributed to the specific management approach that the treating physicians have implemented. Thus, the authors cannot attribute the recovery of the LVF to the management of the DKA. Perhaps such recovery took place as the natural healing process of TTS, while the DKA was being managed. Indeed it is conceivable that the pathophysiological process of TTS is already completed by the time of clinical presentation, and the process of recovery is in motion, rather than such process being a continuing dynamic interaction of catecholamine-induced cardiomyocyte injury affected by comorbid noxious influences (e.g., DKA) or our therapies. Thus, the resolution of TTS, timely linked to the treatment of DKA, can be a coincidence. Unfortunately, we do not possess specific drug management for TTS, and our therapies are merely supportive.
